# Incidence and Survival for Common Cancers Are Lower in New Mexico and Along the US-Mexico Border Than Elsewhere in the United States

**DOI:** 10.7759/cureus.11234

**Published:** 2020-10-29

**Authors:** Zachariah Taylor, Kayla Chory, Mark Wright, Anup Amatya, Charlotte Gard, Michael E Woods

**Affiliations:** 1 Urology, Main Line Health, Philadelphia, USA; 2 Osteopathic Medicine, Burrell College of Osteopathic Medicine, Las Cruces, USA; 3 Radiology, University of Nebraska Medical Center, Lincoln, USA; 4 Economics, Applied Statistics & International Business, New Mexico State University, Las Cruces, USA

**Keywords:** cancer incidence, cancer survival, underserved populations, border region

## Abstract

Background

Few in-depth reports on cancer epidemiology in New Mexico or the United States-Mexico border region exist. We aim to quantify cancer incidence and survival in New Mexico and the United States-Mexico border region in New Mexico.

Methods

Incidence and survival were obtained using SEER*Stat 8.3. The data were divided into either New Mexico, or SEER 18 (comprised of the 17 remaining regions) and then further divided by county in New Mexico and by time period. Incidence rates were age-standardized to the 2000 US census. Five-year survival was calculated for each cancer type. Kaplan-Meier survival plots were produced, and significance was determined using log-rank analysis.

Results

Analysis demonstrated that cancers in New Mexico are diagnosed at a lower rate with the exception of thyroid, liver, and ovarian. Survival is generally lower in New Mexico with 10 of the 14 cancers having worse survival in New Mexico. Only uterine cancer had improved survival in New Mexico (77.9% vs 74.9%, P < .001). Additionally, breast (82.2%), prostate (83.3%), lung and bronchus (13.7%), colorectal (53.7%), melanoma (80.1%), kidney and renal pelvis (61.2%), uterine (78.5%), and ovarian (41.6%) all had lower survival in the border counties.

Conclusion

Comparing New Mexico to the other regions in the SEER 18 database, both cancer incidence and survival are consistently lower; these findings could be explained by lower access to healthcare, which can result in underreporting and delays in diagnosis.

## Introduction

In 1973, the National Cancer Institute (NCI) launched the Surveillance, Epidemiology, and End Results (SEER) program. The program initially collected data from nine states/regions, including New Mexico (NM). The program has since grown to include 18 regions, comprising approximately 34.6% of the United States (US) population [1]. Previous studies using the SEER database have been valuable resources for better understanding incidence, mortality, treatment, and other aspects of cancer in the United States [2,3]. Broad cancer statistics have been observed in NM [4]. However, the SEER cancer data collected from the state of NM have not been extensively analyzed and reported.

NM is one of the four states in the United States that shares a border with Mexico, which creates a unique ethnic demographic. Hispanics and Native Americans represent a larger percentage of NM cancer patients when compared to SEER18, consistent with the demographics of the state. As of July 2016, 48.5% of NM’s population identified as Hispanic or Latino [5], much higher than the 18.3% in the United States population [6]. Given current demographic trends, the US Census Bureau predicts that by the year 2045, non-Hispanic whites will comprise less than 50% of the population, with Hispanics comprising the second-highest percentage [7]. In other words, the future United States will more closely resemble the current demographic features of NM. Understanding current cancer trends and outcomes in NM may help to better understand cancer outcomes in the future United States.

Health disparities in the Hispanic and Latino populations have been well documented [8]. When compared to non-Hispanic whites, Hispanics and Latinos experience greater disease burden, poorer outcomes, and increased mortality due to other comorbidities such as diabetes mellitus [9], cancer [10], liver disease [11], and vascular disease [12].

Cancer incidence and survival data are sparse in NM and are even more lacking in the United States-Mexico border region [13]. Ultimately, our goal is to better understand how cancer incidence and survival in NM differ from what is observed in the other SEER 18 regions, and how the border counties within NM differ from the non-border counties within NM.

## Materials and methods

Registry

Multiple registries of the SEER database exist (SEER 9, SEER 13, SEER 18, and SEER 21, which was added recently in a limited version). Of these registries, the SEER 18 database was used in this study as it was the most comprehensive SEER registry of the United States at the time of the study. The SEER 18 registry includes cancer data from the following states/regions: Alaskan Natives, Atlanta, California, Connecticut, Detroit, greater Georgia, rural Georgia, Hawaii, Iowa, Kentucky, Los Angeles, Louisiana, New Jersey, New Mexico, San Francisco-Oakland, San Jose-Monterey, Seattle-Puget Sound, and Utah. The inclusion of NM and the ability to identify the county of residence for the majority of cancer cases made the SEER 18 the ideal database for this study. Other available cancer databases are less comprehensive or do not contain the same level of detail for NM found in the SEER database. Incidence rates (IR) [14] and five-year survival [15] were obtained using the SEER*Stat 8.3.5 program following the protocols established by the NCI. Observed IRs were obtained for the period 2000-2014 as a whole and for each year during the period for NM and for the other 17 geographical areas of SEER 18 combined (SEER18).

Cancers studied

We analyzed the 14 most common cancers in NM: prostate, breast, lung and bronchus, colorectal, thyroid, melanoma, oral cavity and pharynx, liver and intrahepatic bile duct, kidney and renal pelvis, bladder, pancreatic, uterine, ovarian, and stomach. Incidence and five-year survival for prostate cancer were only analyzed in men, while ovarian and uterine cancers were only analyzed in females. Breast cancer IR and survival include both male and female breast cancer cases.

Statistical analysis

Statistically significant differences in IRs were determined for each cancer based on non-overlapping 95% confidence intervals. However, overlapping does not preclude statistical significance on further testing. We also calculated rate ratios to display differences in cancer IRs between NM and SEER18. Segmented regression models were used to model changes in IR during the study period for each cancer. Two segments from 2000-2007 and 2008-2014 were used and linear regression models were run on both segments. This analysis was used to determine differences in trends between NM and SEER18, simultaneously incorporating changes in trends within each of these two groups during the study period. Slopes derived from segmented regression models were compared for significance using t-tests.

The NM survival data were further segregated into the border and non-border regions. Using the “La Paz” classification set forth by the Baker Institute for Public Health [16], the following counties were grouped together to define the US-Mexico border region: Hidalgo, Luna, Dona Ana, Grant, Sierra, and Otero. The La Paz classification designates that any county that is completely or partially within 100 kilometers from the border be included in the border region. The combined population of California, Arizona, New Mexico, and Texas in the border region is approximately 15 million [17]. In NM, the border population is approximately 350,820, with approximately 1.7 million living in the non-border counties [18]. We observed survival for the NM border counties as compared to those for the remaining (non-border) counties in the state.

Individual-level data for cancer cases were used to generate five-year Kaplan-Meier survival curves and perform log-rank tests to compare survival for NM to SEER18 and for NM border counties to NM non-border counties. An alpha level of 0.05 was used to determine statistical significance. Analyses were conducted using R (version 3.5.1; R Foundation for Statistical Computing, Vienna, Austria) and SAS (version 9.4, SAS Institute, Cary, NC).

Institutional approval

This study was reviewed by the Burrell College of Osteopathic Medicine Institutional Review Board and was designated as exempt (IRB# 00491_2019).

## Results

Incidence

A total of 144,010 patients were studied in New Mexico with 51.7% male and 6,556,496 in SEER 18 with 51.2% male. Of the individuals in New Mexico, 24,971 were located in the border counties with 54.5% male, while 120,300 were located in non-border counties with 51.2% male (Table 1). Analysis of the 14 most common cancers in NM shows that, in general, cancer IRs in NM are significantly lower than in the remainder of SEER18 for the period 2000 to 2014. As seen in Table 2, this is true of all cancers analyzed except for thyroid, liver, and ovarian. Thyroid and liver cancers are both diagnosed at a significantly higher rate in NM. Ovarian cancer has a similar IR in NM as in SEER18.

**Table 1:** Demographic breakdown of cancer totals and insurance status Demographic cancer statistics for the four groups studied.

All Cancer	New Mexico	SEER 18	NM Border Counties	NM Non-Border Counties
Total	144,010	6,556,496	24,971	120,300
Male	74,413 (51.7%)	3,355,295 (51.2%)	13,628 (54.5%)	61,630 (51.2%)
Female	69,597 (48.3%)	3,201,201 (48.8%)	11,343 (55.5%)	58,670 (58.8%)
Uninsured	2,585 (1.8%)	96,821 (1.5%)	585 (2.3%)	2,044 (1.7%)
Any Medicaid	9,062 (6.3%)	443,243 (6.8%)	1,690 (6.8%)	7,521 (6.3%)
Insured	48,810 (33.9%)	2,599,388 (39.6%)	8,153 (32.6%)	41,139 (34.2%)
Insured/No Specifics	15,074 (10.5%)	612,766 (9.3%)	3,586 (14.4%)	11,801 (9.8%)
Insurance status unknown	13,548 (9.4%)	297,420 (4.5%)	1,525 (6.1%)	11,892 (9.9%)
Blanks	54,931 (38.1%)	2,506,858 (38.2%)	9,432 (37.8%)	45,903 (38.2%)

**Table 2:** Cancer incidence in New Mexico from 2000-2014 Number of cases of each cancer type for New Mexico, and SEER18 from 2000-2014. Cancers are ordered based on the total number of cases in New Mexico during the study period. Additionally, the incidence per 100,000 for each cancer is listed with associated 95% confidence intervals. The rate ratio is reported for each cancer. Twelve of the 14 cancers had a rate ratio <1.0, with only ovarian cancer having overlapping 95% confidence intervals. Thyroid and liver and intrahepatic bile duct cancers were the only cancers with rate ratio >1.0. * is representative of overlapping 95% confidence intervals ** is representative of cancers with rate ratio >1.0

Cancer Type	New Mexico (NM) Total Number of Cases	SEER18 Total Number of Cases	NM Incidence per 100,000 (95% CI)	SEER18 Incidence per 100,000 (95% CI)	Rate Ratio
Breast	21,733	979,318	59.9 (59.0-60.7)	68.3 (68.2-68.4)	0.88
Prostate	20,904	924,292	118.8 (117.2-120.5)	141.8 (141.5-142.1)	0.84
Lung and Bronchus	15,997	848,748	44.0 (43.3-44.7)	60.4 (60.3-60.5)	0.73
Colorectal	13,760	637,055	37.9 (37.3-38.6)	45.0 (44.9-45.1)	0.84
Melanoma	6,237	305,600	17.4 (17.0-17.9)	21.5 (21.4-21.6)	0.81
Bladder	5,650	287,079	15.6 (15.2-16.1)	20.6 (20.5-20.6)	0.76
Kidney and Renal Pelvis	4,947	214,802	13.5 (13.2-13.9)	15.0 (14.9-15.1)	0.90
Thyroid**	4,637	169,638	13.5 (13.1-13.9)	11.9 (11.9-12.0)	1.13
Uterine	4,274	194,909	21.9 (21.2-22.6)	24.8 (24.7-25.0)	0.88
Liver and Intrahepatic Bile Duct**	3,913	113,850	10.7** (10.4-11.1)	7.8 (7.7-7.8)	1.37
Pancreatic	3,913	173,151	10.7 (10.4-11.1)	12.3 (12.2-12.3)	0.87
Oral Cavity and Pharynx	3,470	161,165	9.4 (9.1-9.7)	11.1 (11.0-11.1)	0.85
Stomach	2,494	106,836	7.0 (6.7-7.3)	7.6 (7.5-7.6)	0.92
Ovarian*	2,348	96,563	12.2 (11.7-12.7)	12.5 (12.4-12.6)	0.98

The cancer with the highest incidence was prostate with an IR of 118.8 per 100,000 in NM and 141.8 in SEER18, followed by breast (59.9 in NM and 68.3 in SEER18, combined male and female), lung and bronchus (44.0 in NM and 60.4 in SEER18), and colorectal (37.9 in NM and 45.0 in SEER18).

The rate of change in incidence during the study period also differed for some of the cancers studied (data not shown). Breast, prostate, lung and bronchus, colorectal, bladder, stomach, and ovarian cancers displayed a decreasing incidence trend in both the NM data and the SEER18 data. Kidney and renal pelvis, thyroid, uterine, liver and intrahepatic bile duct, and pancreatic cancers displayed an increasing incidence trend in both NM and SEER18. Oral cavity and pharynx cancer displayed an increasing trend in SEER18, but a decreasing trend in NM.

While most cancers followed similar changes in incidence rates (∆IR), segmented regression analysis showed that the ∆IR for a few cancers differed in NM compared to SEER18.

Prostate cancer displayed consistently lower rates in NM throughout the study period; however, in both NM and SEER18 the IR changed dramatically during the study period. Segmented regression analysis revealed that the IR for NM and SEER18 fell at rates of -1.2 and -1.9 per 100,000, respectively, prior to 2007 (data not shown). Since 2007, the IRs for NM and SEER18 have fallen at significantly higher rates compared to prior years (-10.7 and -8.3 per 100,000 for NM and SEER18, respectively). The difference of -2.4 in the rate of decrease in the IR between NM and SEER18 was not statistically significant (P = .07).

The IR for melanoma has declined in NM but is rising for SEER18. From 2000 to 2007, the IR for SEER18 rose at a rate of 0.48 per 100,000 per year. In contrast, the IR in NM decreased at a rate of 1.8 per 100,000 per year. The IR in NM abruptly jumped to 21.2 per 100,000 in 2008 but has fallen sharply since, at the rate of -1.3 per 100,000 per year.

The kidney and renal pelvis cancer IRs in NM and SEER18 were not significantly different in 2000 (P = .66) at approximately 12 per 100,000. However, the rates rose significantly faster for SEER18 (∆IR = 0.64 vs. 0.23 for NM, P = .004) until 2008. At that point, the SEER18 IR had risen to 15.4 per 100,000 and had begun to show signs of leveling off or slowly declining. However, the IR for NM continued to rise with a significant slope of 0.23 per 100,000 per year.

The uterine cancer IR in NM was consistently below than in the SEER18 group; however, SEER18 and NM rates began to rise from 2005, with NM rising relatively faster than SEER18. The IR in NM and SEER18 remained unchanged, until 2005, at 19.7 and 23.3 per 100,000, respectively. The IR rose in both NM and SEER18 after 2005. The IR rose with the rate of 0.69 (95% CI: 0.53, 0.85) in NM and 0.37 (95% CI: 0.21, 0.53) per 100,000 for SEER18. In 2014, the estimated IR was 25.4 (95% CI: 24.4, 26.7) in NM, and 29.9 (95% CI: 24.8, 35.0) in SEER18.

In oral cavity and pharynx cancers, the IR in NM in the year 2000 was -1.5 per 100,000 lower than the IR in SEER18 (P = .0001) with an estimated IR of 9.3 per 100,000 in NM and 10.8 per 100,000 in SEER18. Since 2000, the IRs in NM and SEER18 showed a non-significant increasing trend until 2007. After 2007, the IR in NM exhibited a non-significant decreasing trend (∆IR = -0.09, P = .13). In contrast, the IR in SEER18 showed a non-significant but increasing trend (∆IR = 0.08, P = .17).

The ovarian cancer IR in the SEER18 group has fallen consistently; however, the IR in NM has leveled off since 2010 and may show signs of increasing. From 2000 to 2006, the IR in NM and SEER18 decreased with a significant downward trend. The IR decreased with the rate of -0.31 (95% CI: -0.47, -0.15) in NM and -0.23 (95% CI: -0.39, -0.07) per 100,000 in SEER18. From 2007, the estimated IR in NM has not changed significantly from 12.21 (95% CI: 11.7, 12.8). The IR in SEER18, however, has continued to decline with a rate of -0.24 (95% CI: -0.37, -0.12) per 100,000.

Survival

Next, we analyzed the five-year survival from diagnosis in NM, SEER18, the NM border counties, and the NM non-border counties (Table 3). Five-year survival in NM was generally lower than in SEER18. Of the 14 cancers studied, only uterine cancer had significantly higher survival in NM than in SEER18 (77.9% vs 74.9%, P < .001). Ten cancers displayed significantly lower five-year survival in NM when compared to the SEER18 as demonstrated in Table 3. The five-year survival for breast (P = .5), oral pharynx (P = 0.08), and ovarian cancers (P = .3) were also lower in NM than in SEER18, but these differences were not statistically significant.

**Table 3:** Five-year survival by cancer type Five-year survival for New Mexico, SEER, New Mexico Border Counties, and New Mexico Non-Border Counties with accompanying log-rank test results for New Mexico vs SEER and Border vs Non-Border counties.

Cancer Type	SEER18 Survival	NM Survival	NM Border Counties	NM Non-Border Counties	NM vs SEER18 log-rank P-value	NM Border vs NM Non-Border log-rank P-value
Breast	81.3%	81.1%	76.0%	82.2%	.5	< .001
Prostate	83.9%	82.8%	80.1%	83.3%	< .001	< .001
Lung and Bronchus	14.6%	12.7%	10.9%	13.7%	< .001	< .001
Colorectal	54.7%	52.9%	50.0%	53.7%	< .001	< .001
Melanoma	82.1%	79.3%	73.5%	80.1%	< .001	< .001
Bladder	62.9%	60.6%	60.6%	60.6%	.002	.5
Kidney and Renal Pelvis	63.9%	59.7%	57.1%	61.2%	< .001	.02
Thyroid	93.9%	92.8%	91.4%	93.3%	.04	.5
Uterine	74.9%	77.9%	73.5%	78.5%	< .001	.01
Liver and Intrahepatic Bile Duct	14.5%	11.2%	10.9%	12.9%	< .001	.2
Pancreatic	6.2%	5.7%	4.8%	5.9%	< .001	.6
Oral Cavity and Pharynx	56.3%	55.2%	50.6%	56.3%	.08	.1
Stomach	24.4%	20.7%	19.9%	21.7%	< .001	.9
Ovarian	41.9%	41.6%	35.9%	41.6%	.3	.03

The Kaplan-Meier method revealed that 13 of the 14 cancers had lower five-year survival in the NM border counties than the NM non-border counties (Table 3). The hazard ratio for eight of these cancers was significantly higher when compared using the log-rank test. Thyroid, liver and intrahepatic bile duct, pancreatic, oral cavity and pharynx, and stomach cancers did not have significantly lower five-year survival despite having lower survival. Bladder cancer showed equal five-year survival rates in the border and non-border counties.

## Discussion

The results point to three key findings: 1) NM has lower IRs when compared to SEER18, 2) five-year survival is consistently worse in NM when compared to SEER18, and 3) within NM, the NM border counties have worse five-year survival outcomes when compared to NM non-border counties.

Of the 14 cancers studied, all but two (liver and thyroid) had lower IR in NM when compared to SEER18. Incidence rates can be affected by risk factors that may be present in a population, as well as disease screening. Determining the relative contribution of each of these factors is difficult due to the complex nature of disease risk and the variability of cancer screening.

One possible explanation for the generally lower IR in NM is lower insurance coverage. Since the Affordable Care Act of 2010, the percentage of uninsured New Mexicans has consistently decreased. In 2011, the percentage of uninsured adults in New Mexico was 26.1%, compared to 21.3% nationally. In 2016, the uninsured percentage in New Mexico had decreased to 12.0%, nearly identical to the national average of 12.4% [4]. Previous studies have shown that individuals without insurance have poorer outcomes when diagnosed with cancer, likely due to greater disease progression at the time of diagnosis [19]. Also, individuals with health insurance receive more consistent cancer screening [20]. Thus, the historically lower insurance coverage in NM could account for the lower IR observed.

The only two cancers with higher IR in NM were liver and thyroid. Liver cancer could be explained by the higher rates of liver cirrhosis due to hepatitis C. Nationally, the incidence of hepatitis C has been increasing. The CDC reports an incidence of 1.0 per 100,000 in the US in 2016 [21]; however, the NM hepatitis C virus (HCV) infection incidence rate is increasing at more than double the national rate [22]. In addition to hepatitis C, NM exceeds the national average in alcohol-related chronic liver disease and NM mortality rate from chronic liver disease is more than twice that of the United States [23]. The risk of liver cancer from hepatitis C infection and excessive alcohol consumption has been well documented [11].

Multiple studies have shown that thyroid cancer has a high IR in northern NM and may be linked to prior nuclear weapons development at Los Alamos [24]. In our study, we observed consistently higher IR in NM compared to SEER18. Before the year 2006, the change in incidence rates was greater in NM than SEER18. However, from 2006 to 2014, the change in incidence rates for NM and SEER18 was the same. This would be consistent with fewer individuals with prior high radiation exposure still living.

Our results on five-year survival display a worse cancer prognosis in NM, compared to SEER18. Thirteen of the 14 cancers studied had worse five-year survival in NM. Only uterine cancer had a better prognosis in NM. Insurance coverage and access to health care could be driving forces for the poorer prognosis in NM. It has previously been shown that insurance results in earlier detection. Some of the great achievements in cancer mortality prevention have been due to improved screening, such as in colorectal cancer [25]. These efforts require access to medical professionals trained to perform these screenings accurately. With less insurance coverage and lack of access to health care in NM, it may be possible that NM residents are diagnosed at later stages of the disease. As seen in Table 1, NM has higher rates of uninsured, further supporting the likelihood of insurance status being a contributing factor.

Language barriers may also contribute to differences in cancer statistics in NM. Thirty-five percent of New Mexicans speak a language other than English at home, primarily Spanish [5]. Woloshin et al. showed that language barriers result in decreased compliance by patients and lack of appropriate follow-up [26]. Patients consistently prefer medical care providers who speak their native language [27]. Although the percentage of Spanish speaking physicians in New Mexico is unknown, the relatively large number of non-English speakers in NM may influence the access to and delivery of healthcare in the state.

Thirteen of the 14 cancers studied displayed worse five-year survival in the NM border counties, although the reason for this is unclear. Previous studies into border health are very limited, with no previous studies that the authors are aware of detailing cancer IR and five-year survival in NM. Prior investigations into cancer care in the border region have used surveys to reveal that many individuals report challenges in obtaining proper cancer care [28]. Respondents report challenges in coordinating cancer care that often results in delay of care, and at times cross-border discordance regarding cancer diagnosis and treatment, causing confusion. A regional shortage of cancer specialists, and financial hardship, further exacerbate the challenge of obtaining proper cancer care. To combat this, educational programs have been established to try and provide a foundation for the framework of receiving cancer screening and treatment in the US [29]. There is limited documentation of the success of these programs in the border region.

Strengths and limitations

The SEER 18 registry covers approximately 34% of the US population, and we assume that trends within this dataset are generalizable to the entire US population. One weakness of the SEER database is that SEER oversamples urban areas, which may account for some of the differences noted in our study due to the general rural population of NM. Additionally, the SEER database does not provide any information on comorbidities or other variables inherent in a retrospective study, which therefore cannot be accounted for in the study and may confound some of the results. Cancer treatment is constantly evolving and improving. One weakness of the study is that it does not capture the current state of cancer care and any advances in diagnosis or treatment that may have occurred since 2015.

## Conclusions

Our results show that the cancer incidence rates and five-year survival in New Mexico are significantly different than those found in the remaining SEER 18 database. Incidence is consistently lower in New Mexico when compared to SEER 18, except for liver and thyroid cancers, which have higher incidence rates. Five-year survival is worse in New Mexico, with 10 of the 14 cancers studied being significantly worse. Further analysis revealed that, generally, five-year survival is worse in the border counties than in the non-border counties of New Mexico, with eight of the 14 cancers studied being significantly worse. The driving forces for the differences observed are unknown; however, factors such as access to healthcare, lower insurance rates, and socioeconomics deserve further investigation.

## References

[1] (2020). SEER incidence data, 1975-2017. https://seer.cancer.gov/data/.

[2] Filippou P, Ferguson JE III, Nielsen ME (2016). Epidemiology of prostate and testicular cancer. Semin Intervent Radiol.

[3] Marley AR, Nan H (2016). Epidemiology of colorectal cancer. Int J Mol Epidemiol Genet.

[4] Gallagher L (2018). The State of Health in New Mexico, 2018. Mexico.

[5] (2020). U.S. Census Bureau QuickFacts: New Mexico. https://www.census.gov/quickfacts/nm..

[6] (2020). U.S. Census Bureau QuickFacts: United States. https://www.census.gov/quickfacts/fact/table/US/PST045218.

[7] United States Census Bureau (2018). Older People Projected to Outnumber Children for First Time in U.S. History. https://www.census.gov/newsroom/press-releases/2018/cb18-41-population-projections.html.

[8] Vega WA, Rodriguez MA, Gruskin E (2009). Health disparities in the Latino population. Epidemiol Rev.

[9] Remler DK, Teresi JA, Weinstock RS, Ramirez M, Eimicke JP, Silver S, Shea S (2011). Health care utilization and self-care behaviors of medicare beneficiaries with diabetes: comparison of national and ethnically diverse underserved populations. Popul Health Manag.

[10] Stefanidis D, Pollock BH, Miranda J (2006). Colorectal cancer in Hispanics: a population at risk for earlier onset, advanced disease, and decreased survival. Am J Clin Oncol.

[11] El-Serag HB, Mason AC (2000). Risk factors for the rising rates of primary liver cancer in the United States. Arch Intern Med.

[12] Morrissey NJ, Giacovelli J, Egorova N (2007). Disparities in the treatment and outcomes of vascular disease in Hispanic patients. J Vasc Surg.

[13] Taylor ZD, McLeod E, Gard CC, Woods ME (2020). Testicular cancer incidence and mortality in New Mexico. Ethn Dis.

[14] (2020). SEER*Stat tutorials: basic incidence & trends. https://seer.cancer.gov/seerstat/tutorials/basic.html.

[15] (2020). SEER*Stat tutorials: survival & left truncated statistics. https://seer.cancer.gov/seerstat/tutorials/survival.html.

[16] Payan T, Cruz PL (2017). Managing the U.S.-Mexico Border First Requires Defining It. https://www.bakerinstitute.org/media/files/files/fc064f03/BI-Brief-042017-MEX_DefineBorder.pdf.

[17] Affairs (OGA) O of G (2020). The U.S.-Mexico border region. https://www.hhs.gov/about/agencies/oga/about-oga/what-we-do/international-relations-division/americas/border-health-commission/us-mexico-border-region/index.html.

[18] United States Census Bureau (2020). Population and Housing Unit Estimates. https://www.census.gov/programs-surveys/popest.html.

[19] Niu X, Roche LM, Pawlish KS, Henry KA (2013). Cancer survival disparities by health insurance status. Cancer Med.

[20] Hsia J, Kemper E, Kiefe C (2000). The importance of health insurance as a determinant of cancer screening: evidence from the women’s health initiative. Prev Med.

[21] (2019). Surveillance for viral hepatitis - United States, 2016. https://www.cdc.gov/hepatitis/statistics/2016surveillance/index.htm.

[22] Campbell CA, Canary L, Smith N, Teshale E, Ryerson AB, Ward JW (2017). State HCV incidence and policies related to HCV preventive and treatment services for persons who inject drugs — United States, 2015-2016. Am J Transplant.

[23] (2020). Substance abuse epidemiology. https://nmhealth.org/about/erd/ibeb/sap/.

[24] Udelsman R, Zhang Y (2014). The epidemic of thyroid cancer in the United States: the role of endocrinologists and ultrasounds. Thyroid.

[25] Kahi CJ, Imperiale TF, Juliar BE, Rex DK (2009). Effect of screening colonoscopy on colorectal cancer incidence and mortality. Clin Gastroenterol Hepatol.

[26] Woloshin S, Bickell NA, Schwartz LM, Gany F, Welch HG (1995). Language barriers in medicine in the United States. JAMA.

[27] Diamond LC, Luft HS, Chung S, Jacobs EA (2012). “Does this doctor speak my language?” Improving the characterization of physician non-English language skills. Health Serv Res.

[28] Ko E, Zúñiga ML, Palomino H, Peacher D, Watson M (2018). Qualitative study of Latino cancer patient perspectives on care access and continuity in a rural, U.S.-Mexico border region. J Immigr Minor Health.

[29] Palomino H, Peacher D, Ko E, Woodruff SI, Watson M (2017). Barriers and challenges of cancer patients and their experience with patient navigators in the rural US/Mexico border region. J Cancer Educ.

